# A short-term forecasting method for photovoltaic power generation based on the TCN-ECANet-GRU hybrid model

**DOI:** 10.1038/s41598-024-56751-6

**Published:** 2024-03-21

**Authors:** Xiuli Xiang, Xingyu Li, Yaoli Zhang, Jiang Hu

**Affiliations:** 1 School of Economics, Wuhan Textile University, Wuhan, 430077 China; 2 Zhongnan University of Economics and Law, Wuhan, 430073 China; 3 School of Economics and Management Organization, Hubei University of Automotive Technology, Shiyan, 442002 China

**Keywords:** Short-term, Photovoltaic power forecasting, TCN, ECANet, GRU, Energy science and technology, Engineering

## Abstract

Due to the uncertainty of weather conditions and the nonlinearity of high-dimensional data, as well as the need for a continuous and stable power supply to the power system, traditional regression analysis and time series forecasting methods are no longer able to meet the high accuracy requirements of today's PV power forecasting. To significantly improve the prediction accuracy of short-term PV output power, this paper proposes a short-term PV power forecasting method based on a hybrid model of temporal convolutional networks and gated recurrent units with an efficient channel attention network (TCN-ECANet-GRU) using the generated data of an Australian PV power station as the research object. First, temporal convolutional networks (TCNs) are used as spatial feature extraction layers, and an efficient channel attention network (ECANet) is embedded to enhance the feature capture capability of the convolutional network. Then, the GRU is used to extract the timing information for the final prediction. Finally, based on the experimental validation, the TCN-ECANet-GRU method generally outperformed the other baseline models in all four seasons of the year according to three performance assessment metrics: the normalized root mean square error (RMSE), normalized mean absolute error (MAE) and coefficient of determination (R^2^). The best RMSE, MAE and *R*^2^ reached 0.0195, 0.0128 and 99.72%, respectively, with maximum improvements of 11.32%, 8.57% and 0.38%, respectively, over those of the suboptimal model. Therefore, the model proposed in this paper is effective at improving prediction accuracy. Using the proposed method, this paper concludes with multistep predictions of 3, 6, and 9 steps, which also indicates that the proposed method significantly outperforms the other models.

## Introduction

Along with the escalating conflict between environmental protection and growing energy demand, clean energy is gradually gaining worldwide attention. Among them, photovoltaic power generation, as a type of clean energy, is constantly being popularly used due to its advantages, such as safety, extensiveness, sufficiency, and potential economy. However, photovoltaic power generation is susceptible to intermittent and unstable power generation due to factors such as climatic features and the alternation of day and night^1^, which leads to difficulties in matching the demand and supply to the grid and poses certain scheduling challenges for the power system. Short-term forecasting of PV power, therefore, contributes to timely coordination of the power system, reduces the impact of fluctuations in PV power on the grid, and provides the basis for a continuous and stable power supply and demand.

At present, photovoltaic power generation forecasting methods can be roughly divided into statistical methods, traditional machine learning methods, and deep learning methods. Statistical methods include linear regression, ARMA time series analysis, and the Markov chain model^2^. Although the statistical methods are highly explanatory, the model is complex when considering the many influencing factors, which is not conducive to practical forecasting and often results in large prediction errors when dealing with sudden changes in PV power.

Machine learning methods include a probabilistic neural network (PNN) used to divide subsets of different weather types, principal component analysis (PCA) for dimensionality reduction, and support vector regression (SVR) optimized by the scattered search algorithm (SS) to forecast the short-term output of PV power generation, as proposed by Wang Xin et al.^3^. Song et al.^4^ proposed a combined prediction model consisting of multiple regression trees, which possesses good prediction performance compared with a variety of traditional methods. Wang et al.^5^ used a gradient boosting decision tree (GBDT) to train on historical weather data and historical power generation data, and the resulting prediction models had the advantages of strong interpretability and stable error performance. Massaoudi M et al.^6^ performed feature selection based on Bayesian ridge regression (BRR), decomposed the feature data using a continuous wavelet transform (CWT), and finally predicted PV power generation via the CatBoost algorithm. CatBoosting performed well for categorical feature processing, and the model performed better in terms of the coefficient of determination and actual error. Traditional machine learning methods often require manual feature extraction to obtain good results before work, but it is difficult to ensure the versatility of feature extraction in the early stage when facing complex problems. Mahmud et al.^7^ tried to provide and compare the performance of different machine learning algorithms in short-term power generation prediction and long-term prediction of PVs. Mas'ud^8^ compared the performances of KNN, MLR and decision tree regression (DTR) in predicting the hourly PV output power in Saudi Arabia and concluded that KNN is the best.

The deep learning methods applied for photovoltaic power generation forecasting include BP, LSTM, GRU, and Elman neural networks. Zhang et al.^9^ used a 3-layer BP neural network to learn from historical data, and the model's predictions were highly accurate. Zhang et al.^10^ classified the dataset by day type and built an Elman neural network to predict PV power generation. The model has a faster calculation speed and higher prediction accuracy than does the FNN. In 2015, Ye et al.^11^ fed historical power generation, solar radiation intensity, and temperature data into a GA algorithm-optimized fuzzy radial basis function network (RBF) to predict power generation. Hossain and Mahmood^12^ mainly discussed the effect of different input sequence lengths on the performance of single-step prediction models. Considering the characteristics of wind speed, module temperature, ambient and solar radiation, Akhter et al.^13^ constructed an RNN-LSTM model to predict PV power generation for the next 1 h using data from three different PV power stations with a temporal resolution of 5 min. Li et al.^14^ combined wavelet packet decomposition (WPD) to decompose photoelectric sequences into multiple stable subsequences; subsequently, the sequences were fed into LSTM for parallel prediction; the respective outputs were linearly weighted to obtain the final predicted values; this approach outperformed the MLP and RNN methods in terms of MBE, MAPE, and RMSE performance metrics. Bi et al.^15^ proposed a PCNN-BiLSTM model based on CEEMDAN, SSD, and VMD for use as a PV power prediction model; additionally, the decomposed subsequences were fed into parallel convolutional networks and BiLSTM to learn the features; this method ultimately led to improved prediction performance. Yan et al.^16^ utilized frequency domain decomposition and a CNN to extract features and predictions, and the experiments proved that compared with RNNs and long short-term memory (LSTM), CNNs have a significant advantage in terms of time efficiency. Zhou et al.^17^ introduced CEEMDAN signal decomposition and the multiobjective chameleon swarm algorithm to optimize different deep learning methods and investigate the prediction performance of multiple methods on different PV datasets. Zhou et al.^18^ entered separate LSTMs for meteorological and photovoltaic data for prediction and assigned different weights to the data at different time steps via an attention mechanism to focus adaptively on important information; subsequently, the outputs were processed by the flattening layer and combined to obtain the predicted values. The model performed stably in comparison with the ARIMAX and traditional LSTM models for different seasons and time steps.

In recent years, due to the complexity of time series and the limitations of single models, hybrid models that combine convolutional neural networks (CNNs) and recurrent neural networks, such as CNN-LSTM^19,20^, LSTM-CNN^21^ and CNN-GRU^22^, have been widely used; all of these models have good capabilities for PV power forecasting, where the CNN is used mainly for feature extraction of all the attributes and the LSTM and GRU are used for processing features for forecasting. However, ordinary convolutional networks require deeper layers when capturing information over long periods due to the constant size of the convolutional kernel, which tends to cause problems such as gradient disappearance and can directly affect the prediction results. Therefore, Bai et al.^23^ proposed the temporal convolutional network (TCN) in 2017. TCNs can process multiple time series of information in parallel and reduce the consumption of computational resources compared to CNNs and recurrent neural networks.

In this paper, a TCN is used to extract the temporal and correlation information of features. Although the TCN itself has a large receptive field, it is still limited by its convolution kernel size, and the efficient channel attention (ECANet) mechanism is embedded to improve TCN performance. Based on this, this paper proposes a hybrid model of TCN-ECANet-GRU, which learns the spatial features of several historical weather variables and PV power sequences via the improved TCN module of ECANet. Then, the GRU acquires the sequence of time features and establishes the relationship between the features and outputs to predict the PV power. Therefore, the contributions of this paper can be summarized as follows:

1. We propose TCN-ECANet-GRU, which is a newer method for predicting short-term PV power values. This approach represents a relatively new attempt in the field and may also be used for other time series forecasting.
2. The proposed TCN-ECANet-GRU model uses a temporal convolutional network for spatial feature learning from multiple time series to capture temporal and spatial features; on this basis, ECANet is used to improve the performance of TCNs to expand the sensory field of TCNs for better feature capture and prediction.
3. The performance of the proposed TCN-ECANet-GRU method in terms of single-step prediction and multistep prediction is demonstrated based on a case study of a real PV power generation dataset, where the RMSE, MAE and R^2^ are used as evaluation metrics.

## Methods

### Temporal convolutional neural network

A temporal convolutional neural network (TCN) is a neural network structure consisting of a dilated causal convolution structure combined with a residual connection and is mostly used for time series modeling. Dilated causal convolution differs from normal convolution in that "dilated" means that the convolution process is allowed to sample the input data at intervals. The sampling rate $d$ generally increases exponentially with the number of layers; for example, $d=1$ means that each data point is sampled, and $d=2$ means that one data point is sampled every 2 points. Dilated causal convolution also prevents data leakage, where "causal" means that the current moment relates only to the current and past states and contains no information about future sequences. The dilated causal convolution structure of the TCN allows it to have a larger perceptual field than the CNN with fewer layers and fewer parametric numbers, facilitating the reception and processing of longer multiple sequences of information. TCNs are generally formed by stacking multiple residual blocks, each containing two layers of dilated causal convolution, weight normalization, the ReLU activation function, and dropout, and introducing residual connections for direct mapping of the inputs.

### Efficient channel attention mechanism

Channel attention mechanisms for convolutional blocks can improve convolution performance. The efficient channel attention (ECANet) module^24^ avoids the side effects of channel attention caused by the dimensionality reduction of the input and reduces the computational complexity of capturing all channel dependencies compared to the SENet module. It appropriately captures the local cross-channel information interaction with $k$ channels close to the current channel and adaptively calculates and adjusts the size of $k$. The calculation formula for $k$ is shown in Eq. (1):

1 $$\begin{array}{c}k={\left|\frac{{{\text{log}}}_{2}\left(c\right)}{\gamma }+\frac{b}{\gamma }\right|}_{odd}\end{array}$$

where $odd$ is the nearest odd number, $c$ is the current number of channels, $\gamma =2$, and $b=1$.

**Figure 1:**
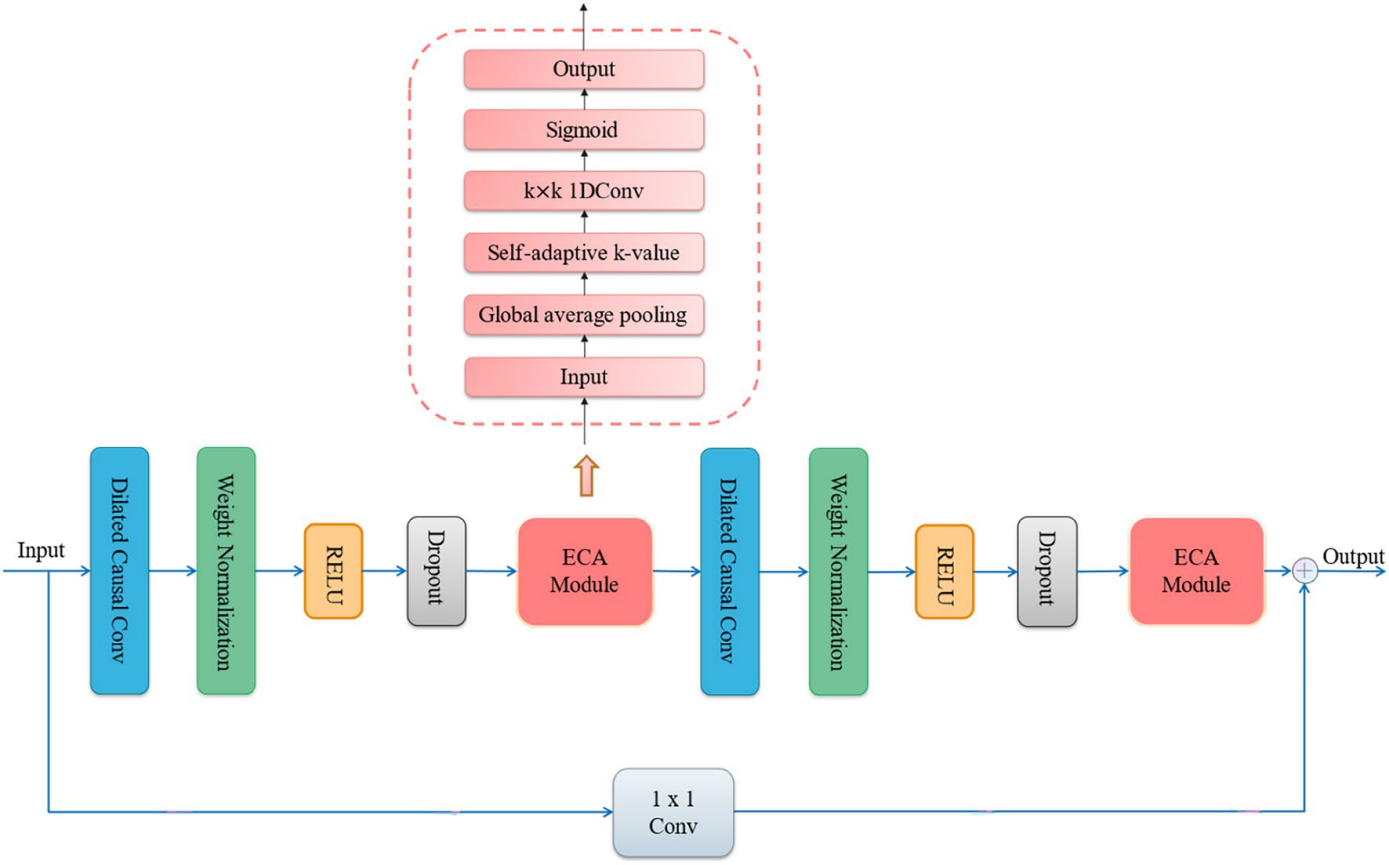
A resblock of TCN.

The TCN-ECA neural network framework consists of a TCN residual block embedded in two ECA modules, as shown in Fig. 1. The ECA module is added after the two-layer dilated causal convolution, and the ECA module performs global average pooling of the input features and generates new channel weights through a one-dimensional convolution layer with a convolution kernel size of $k$, which is activated by $Sigmoid$ and weighted original features for fusion output.

### Gated recurrent unit

The gated recurrent unit network (GRU)^25^ is a variant of LSTM. It merges the LSTM's original input gate and forgotten gate as an update gate, which acts on useful information at the current moment and the previous moment and passes downward. The reset gate controls how much past information needs to be forgotten. Both GRU and LSTM can solve the problem of gradient disappearance caused by RNNs because the input sequence is too long. Compared with LSTM, a GRU has a more concise structure and fewer parameter calculations, and the prediction effect is generally similar to that of LSTM.

### Model framework

**Figure 2:**
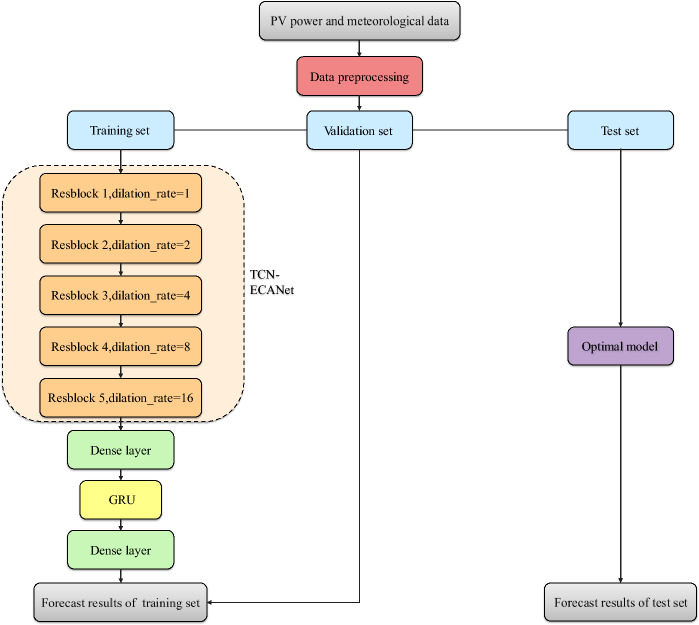
The framework of TCN-ECANet-GRU model.

In this paper, a TCN-ECANet-GRU model for short-term PV power generation forecasting is established. The TCN-ECA module extracts temporal and spatial features of multivariate time series. The GRU further learns the temporal characteristics and establishes the connection between the features and the output to predict photovoltaic power generation. The model framework of this paper is shown in Fig. 2.

### Research framework

**Figure 3:**
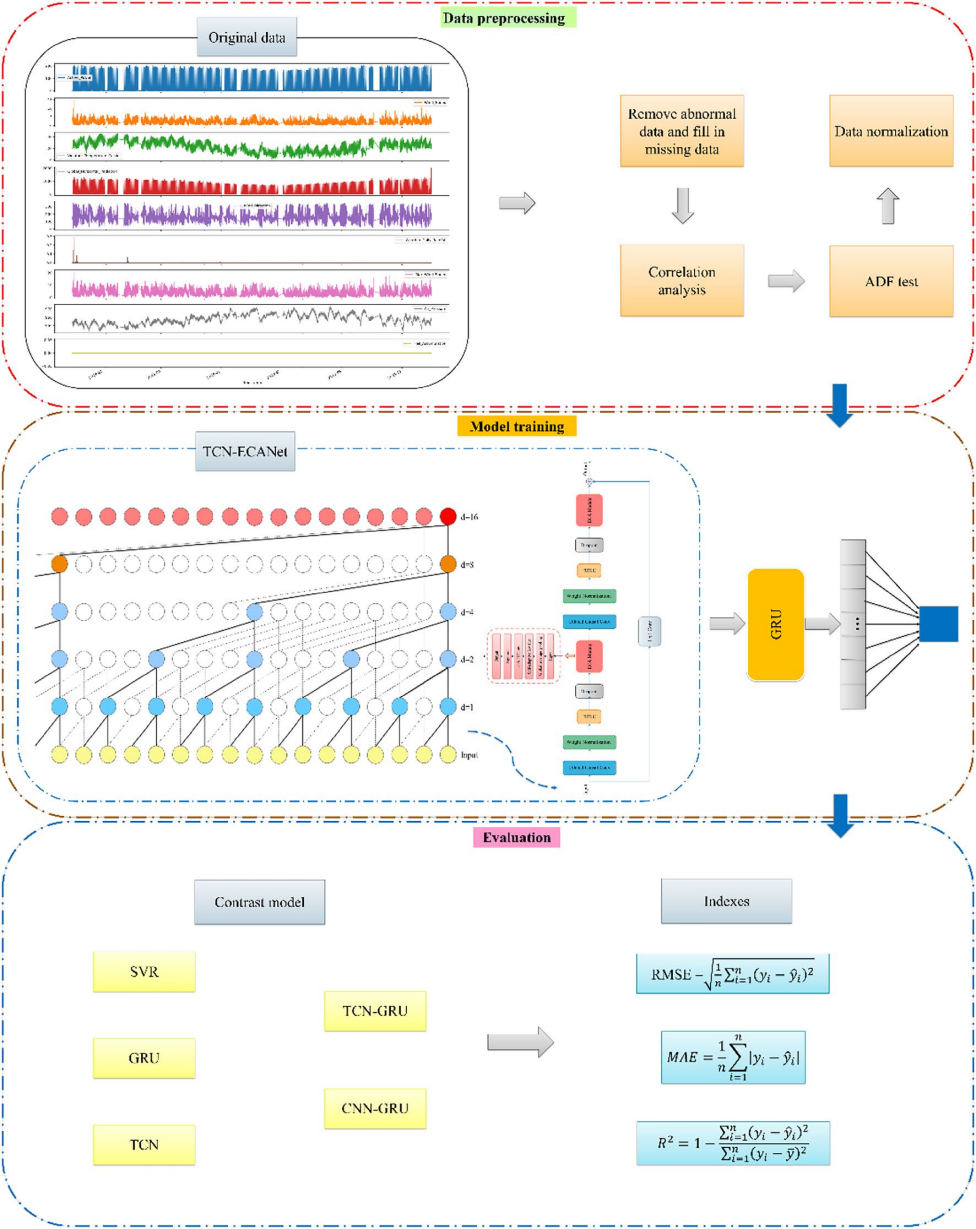
The framework of research.

Figure 3 shows the data visualization and the overall research for the framework. First, data preprocessing, such as missing value processing and normalization, is carried out on the original dataset. Then, the TCN-ECANet-GRU model is employed to predict the next moment of PV power generation. Finally, the RMSE and other evaluation indices are employed to compare the prediction performances of the proposed model and common predictive models. This paper uses real data from the Australian DKASC photovoltaic power station to carry out simulation experiments.

## Data sources and preprocessing

### Data sources

The experimental data used in this paper are from a photovoltaic power dataset (https://dkasolarcentre.com.au/download?location=yulara) from a DKASC photovoltaic power station in Australia. The dataset includes photovoltaic active power data from December 2018 to November 2019 and eight weather features: wind speed, Celsius temperature, wind direction, global horizontal radiation, weather daily rainfall, maximum wind speed, air pressure, and hail accumulation. The temporal resolution is 5 min, which means that the time of day is divided into 288 sampling points, for a total of 95,904 pieces of data. The experiment in this paper predicts the photovoltaic power output at the next moment through all the information in the previous $t$ moments and makes a univariate single-point prediction.

### Correlation analysis

In this paper, we use the Pearson correlation^16^ coefficient to analyze the dataset feature correlation (Eq. (2)).

2 $$\begin{array}{c}{\rho }_{X,Y}=\frac{N\sum XY-\sum X\sum Y}{\sqrt{N\sum {X}^{2}-{\left(\sum X\right)}^{2}}\sqrt{N\sum {Y}^{2}-{\left(\sum Y\right)}^{2}}}\end{array}$$

**Figure 4:**
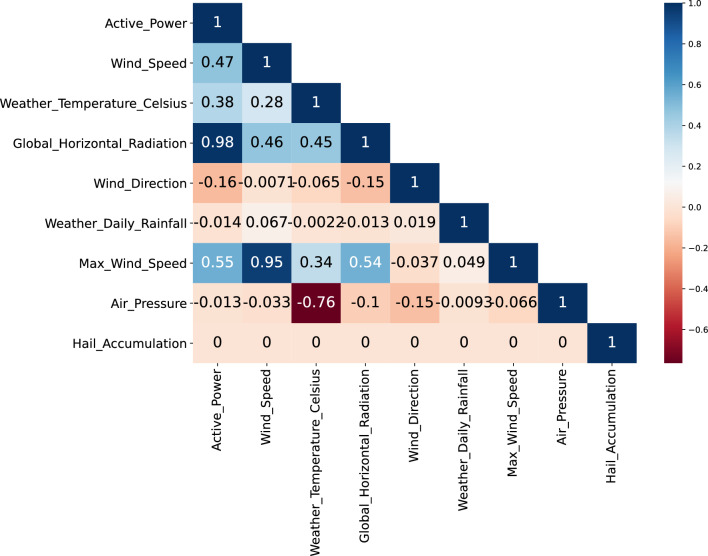
Correlation coefficient of variables.

The degree of correlation between the individual features was analyzed, as shown in Fig. 4. Power generation has relatively strong correlations with global horizontal radiation, maximum wind speed, wind speed and Celsius temperature, but wind direction, weather, daily rainfall and air pressure have little influence on power generation. Since this dataset shows that the hailfall record value is 0 throughout the year, this paper excludes this variable and retains all the other variables to provide as much valid information as possible for the time series forecasting of photovoltaic power generation. In addition, a convolutional neural network is used to grasp the potential connections between features and perform feature fusion, which reflects the learning ability and utilization of multiple features by the hybrid neural network.

### Missing value handling

Due to delays and gaps in the reception and execution of data by power station equipment, the dataset contains many missing values. In this paper, the experiment deletes the data of a full day with more consecutive missing values and uses linear interpolation to complete the missing value filling for other cases. After the missing values are processed, a dataset with 288 complete historical data points per day is obtained.

### Outlier handling

Outliers can have a large negative impact on neural network learning; therefore, before training the model, finding and replacing the outliers of the sequence are needed. This paper adopts the $3\sigma$ principle if the series follows a normal distribution. According to Eqs. (3)–(4), data that differ by more than 3 standard deviations from the sample mean are considered outliers, and the outliers are replaced with data from the moment before the outlier.

3 $$\begin{array}{c}\widetilde{x}=\frac{1}{n}\sum_{i=1}^{n}{x}_{i}\end{array}$$

4 $$\begin{array}{c}{\sigma }^{2}=\frac{1}{n-1}\sum_{i=1}^{n}{\left({x}_{i}-\widetilde{x}\right)}^{2}\end{array}$$

where ${x}_{i}$ is the power generated at the ith time of day and $n$ is the total number of samples in the day.

### ADF test

This paper studies a time series forecasting problem, so the series should be tested for stationarity. The stationarity of a time series requires that the behavior of the time series does not change over time, and it portrays the invariance of the statistical properties of the series over time displacement. An important point in the study of time series is the expectation that the historical data of the series will be used to obtain predictions of its future. Therefore, when historical data properties in a time series remain constant in the future, the forecasting model tends to be reliable and yield good results. Conversely, if the time series is nonstationary, forecasting the future from statistical properties obtained directly from historical data may carry the risk of unreliability^26,27^. Therefore, the ADF test, a common test method for time series stationarity, is used in this paper. Since the experiment divided the yearly data into four seasons for forecasting, the data from each season needed to be tested separately (Table 1).

**Table 1:** Results of the ADF test.

Season	$t$-statistic	P value	Lags used	AIC
Summer	− 21.6216	0.0	46	167,631.4766
Autumn	− 20.7548	0.0	48	170,548.6715
Winter	− 20.1275	0.0	46	145,263.6486
Spring	− 20.1316	0.0	47	168,553.5392

The test results reveal that all four seasonal series are stationary sequences, and the maximum delay order is 46.

### Experimental configuration

After normalizing the seasonal datasets via MinMaxScaler, the datasets are reconstructed via a sliding window. The delay order is set to 46, all the feature information of the past 46 sampling moments is used to form an input matrix, and the photovoltaic power generation power at the next moment is used to predict the output matrix. The training set and test set for each season were divided at a ratio of 9:1, and the results are shown in Table 2. For each training, 20% of the portion from the training set is used for validation, and this part is not included in the training.

**Table 2:** Training sets and test sets in different seasons.

Dataset	Number	Training set	Test set
Summer	22,752	2018-12-01 00:00 to 2019-02-19 23:55	2019-02-20 00:00 to 2019-02-28 23:55
Autumn	24,768	2019-03-01 00:00 to 2019-05-23 23:55	2019-05-24 00:00 to 2019-05-31 23:55
Winter	25,056	2019-06-01 00:00 to 2019-08-22 23:55	2019-08-23 00:00 to 2019-08-31 23:55
Spring	23,328	2019-09-01 00:00 to 2019-11-22 23:55	2019-11-23 00:00 to 2019-11-31 23:55

In this paper, the model framework and parameters are set up mainly based on the literature^28–30^ and adjusted through multiple experimental tests and personal experience. The TCN framework composed of 5 stacked TCN-ECA modules is used in this experiment. There are 32 filters in each module, and the dilation coefficient is^1,2,4,8,16^. The convolution kernel size is 2, and the dropout rate is set to [0.2,0.2,0.2,0.1] depending on the variability of the different seasonal data. A fully connected layer of 16 neurons is spliced behind the TCN framework to enhance feature propagation. The GRU layer has 32 neurons and is followed by a fully connected layer with 10 neurons. Finally, a dropout layer with a dropout rate of 0.2 is set, and the leaky ReLU activation function outputs a predicted value. Adam was selected to optimize the network, the learning rate was set to 0.001, the MSE was the loss function, and the number of epochs was 30.

## Experiment and analysis

### Evaluation metrics

Considering that PV power may vary in magnitude, the root mean square error (RMSE) and mean absolute error (MAE)^31^ can vary in magnitude due to the order of magnitude variation in power. To eliminate the effects of different scales, the normalized root mean square error (RMSE), the normalized mean absolute error (MAE), and the coefficient of determination (R^2^) are introduced as metrics to show the accuracy of the prediction results in this experiment. A smaller RMSE and MAE of the model and a larger i ${R}^{2}$ indicate that the model is better at forecasting. The above evaluation metrics are calculated as shown in Eqs. (5)–(7):

5 $$\begin{array}{c}RMSE=\sqrt{\frac{1}{n}\sum_{i=1}^{n}{\left({y}_{i}-{\widehat{y}}_{i}\right)}^{2}}\end{array}$$

6 $$MAE=\frac{1}{n}\sum_{i=1}^{n}\left|{y}_{i}-{\widehat{y}}_{i}\right|$$

7 $$\begin{array}{c}{R}^{2}=1-\frac{\sum_{i=1}^{n}{\left({y}_{i}-{\widehat{y}}_{i}\right)}^{2}}{\sum_{i=1}^{n}{\left({y}_{i}-\overline{y }\right)}^{2}}\end{array}$$

where ${y}_{i}$ is the normalized actual value, ${\widehat{y}}_{i}$ is the normalized predicted value, $\overline{y }$ is the mean value, and $n$ is the number of samples.

### Results

**Figure 5:**
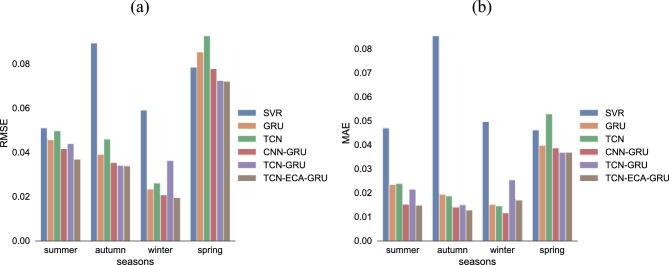
Comparison of RMSE and MAE of models in different seasons: (**a**) RMSE; (**b**) MAE.

**Figure 6:**
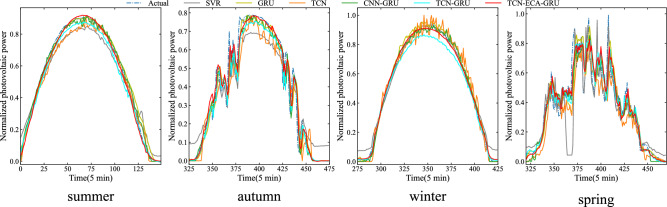
Forecast curves of different models in four seasons.

To verify the feasibility of the proposed model, this paper uses preprocessed data and conducts simulation experiments on a computer. Configuration: System: Win10; CPU: Intel core i7; code language: Python version 3.8; network structure: TensorFlow (version 2.2.0) and Keras (version 2.3.0); editor: VS Code. The TCN-ECANet-GRU model was experimentally tested, and its prediction results were compared with those of three single models, SVR, GRU, and TCN, and two hybrid models, CNN-GRU and TCN-GRU, to evaluate the performance of the models. The predictive performance and true value versus predicted value curves for each model are shown in Table 3, Figs. 5 and 6 (the optimal performance data have been bolded, and space is limited to showing only part of the curve).

**Table 3:** Comparison of RMSE, MAE and R^2^ of models in different seasons.

Metrics	Model	Summer	Autumn	Winter	Spring
RMSE	SVR	0.0509	0.0892	0.0590	0.0783
	GRU	0.0456	0.0390	0.0234	0.0852
	TCN	0.0496	0.0459	0.0261	0.0925
	CNN-GRU	0.0415	0.0352	0.0208	0.0776
	TCN-GRU	0.0438	0.0339	0.0362	0.0723
	TCN-ECANet-GRU	**0.0368**	**0.0338**	**0.0195**	**0.0719**
MAE	SVR	0.0469	0.0853	0.0496	0.0461
	GRU	0.0235	0.0194	0.0151	0.0398
	TCN	0.0238	0.0187	0.0145	0.0529
	CNN-GRU	0.0152	0.0140	**0.0117**	0.0387
	TCN-GRU	0.0215	0.0149	0.0254	**0.0367**
	TCN-ECANet-GRU	**0.0148**	**0.0128**	0.0170	0.0368
R^2^	SVR	0.9714	0.8575	0.9699	0.9298
	GRU	0.9822	0.9833	0.9964	0.9221
	TCN	0.9779	0.9786	0.9954	0.8883
	CNN-GRU	0.9848	0.9865	0.9970	0.9294
	TCN-GRU	0.9827	0.9874	0.9888	0.9379
	TCN-ECANet-GRU	**0.9886**	**0.9885**	**0.9972**	**0.9411**

The bolded data in the table are the optimal results under the same conditions.

Photovoltaic power generation is influenced mainly by solar radiation and shows strong cyclicality. Generally, between 19:00 on the same day and 6:00 on the following day, the power generation is zero and reaches its highest point between midday and afternoon, with some differences between the four seasons. Figure 6 shows that the predictions of the TCN-ECANet-GRU model fit the true values well and are more sensitive to frequent fluctuations and abrupt change points. In contrast, its prediction curve in autumn shows nonzero power generation in the evening, probably because solar radiation is zero in the evening, when minor changes in other weather features can have a more pronounced effect, ultimately making the power prediction nonzero, which also results in a slightly higher MAE value than that of some single models in that season. As shown in Table 3 and Fig. 5, the model proposed in this paper has good forecasting performance, with a significant decrease in the error evaluation metric for each seasonal forecast. The prediction performance of the hybrid models in any season is better than that of the single models because the hybrid models pay more attention to the spatial features between multiple series while considering the temporal features of the series and have a better ability to predict the fluctuations and peak points of the power generation sequence. TCN-GRU has 3.69% and 6.83% lower RMSEs and 0.09% and 0.91% greater R^2^ than CNN-GRU in summer and winter, respectively, which shows that TCN has a larger field of perception than CNN and can capture more information between sequence features. In addition, TCN-ECANet-GRU outperformed the other models in terms of RMSE and R^2^ in spring, summer and winter, with the RMSE decreasing by 15.98%, 0.29% and 0.55%, respectively, and R^2^ improving from 98.88% to 99.72%, respectively, at the highest compared to those of TCN-GRU. It is shown that embedding a 2-layer ECA module into the TCN to assign different weights to the channels effectively improves the ability of the TCN to capture spatial and temporal features and achieve better feature extraction. The model has the highest prediction accuracy in autumn and a slightly inferior prediction performance in winter compared to the other seasons. The main reason for this difference is shown in Fig. 6. The power generation series is more stable in winter, showing more linearity and cyclicity, and the model is easy to train and obtains good results. There are large and high-frequency fluctuations in the power series during the spring months, so the prediction accuracy of the model is slightly reduced, but the model proposed in this paper still outperforms the other models in this respect.

Because the TCN captures the maximum potential connection between power generation and weather conditions, the ECA module captures valid information precisely and focuses on important information from it. Then, the GRU acquires temporal features effectively, taking full advantage of the convolutional neural network to extract spatial features and recurrent neural networks to extract temporal features and providing a feasible solution for short-term prediction of multivariate PV power generation containing factors such as weather. Combined with Table 3 and Figs. 5 and 6, it can be illustrated that the TCN-GRU model embedded in the ECA module effectively improves the prediction performance, has higher prediction accuracy, and can achieve better power generation forecasting results. The robustness of the proposed model is also verified by prediction experiments conducted during multiple seasons.

### Multistep predictive analytics

To further satisfy the practical application of short-term forecasting and to verify the usability of the model for forecasting in the forward steps, this paper investigates the forecasting performance of the model for forecasting in the forward 3, 6, and 9 steps (15 min, 30 min, 45 min), respectively, using RMSE and MAE as the evaluation metrics (see Tables 4 and 5).

**Table 4:** The RMSE for multistep prediction with different models.

Season	Step	SVR	GRU	TCN	CNN-GRU	TCN-GRU	TCN-ECANet-GRU
Summer	3	0.0731	0.0557	0.0591	0.0553	0.0539	0.0518
	6	0.0790	0.0700	0.0789	0.0616	0.0572	0.0563
	9	0.0845	0.0805	0.0767	0.0730	0.0693	0.0669
Autumn	3	0.0821	0.0558	0.0536	0.0581	0.0504	0.0409
	6	0.0836	0.0713	0.0693	0.0691	0.0512	0.0482
	9	0.0865	0.0854	0.0700	0.0749	0.0670	0.0511
Winter	3	0.0666	0.0271	0.0432	0.0412	0.0521	0.0272
	6	0.0684	0.0399	0.0377	0.0497	0.0534	0.0322
	9	0.0703	0.0439	0.0403	0.0548	0.0828	0.0393
Spring	3	0.1092	0.1068	0.0973	0.1078	0.0958	0.0888
	6	0.1138	0.1102	0.1059	0.1098	0.1043	0.0950
	9	0.1165	0.1248	0.1184	0.1232	0.1047	0.1055

**Table 5:** MAEs for multistep predictions with different models.

Season	Step	SVR	GRU	TCN	CNN-GRU	TCN-GRU	TCN-ECANet-GRU
Summer	3	0.0600	0.0296	0.0320	0.0286	0.0363	**0.0273**
	6	0.0650	0.0400	0.0469	0.0317	0.0333	**0.0302**
	9	0.0680	0.0452	0.0392	**0.0345**	0.0487	0.0365
Autumn	3	0.0729	0.0362	0.0273	0.0334	0.0366	**0.0222**
	6	0.0735	0.0536	0.0377	0.0320	0.0283	**0.0219**
	9	0.0753	0.0622	0.0437	0.0419	0.0522	**0.0234**
Winter	3	0.0611	**0.0158**	0.0264	0.0245	0.0399	0.0209
	6	0.0624	0.0246	0.0220	0.0288	0.0399	**0.0201**
	9	0.0639	0.0262	**0.0209**	0.0317	0.0620	0.0267
Spring	3	0.0854	0.0529	**0.0460**	0.0550	0.0642	0.0467
	6	0.0881	0.0600	0.0563	0.0572	0.0665	**0.0524**
	9	0.0893	0.0675	0.0630	0.0628	0.0724	**0.0608**

The bolded data in the table are the optimal results under the same conditions.

According to Tables 4 and 5, through fine-tuning, the proposed TCN-ECANet-GRU model performs very well in almost all the seasons and all the steps. As the number of prediction steps increases, there is a general trend toward decreasing prediction performance for all the models, probably because further multistep prediction is accompanied by the accumulation of prediction errors. Observing all the evaluation metrics, TCN-ECANet-GRU generally outperforms TCN-GRU under the multistep prediction task, which suggests that the efficient channel attention mechanism can indeed enhance the performance of the TCN in capturing the temporal and spatial relationships of multivariate sequences. In the 3-step prediction, TCN-ECANet-GRU has maximum reductions of 18.85% and 18.68% in terms of the RMSE and MAE, respectively, compared to those of the suboptimal model. In the 6-step prediction, the RMSE and MAE are maximally reduced by 8.92% and 22.61%, respectively. In the 9-step prediction, the maximum reductions in the RMSE and MAE reach 23.73% and 44.15%, respectively, which to some extent indicate that the proposed model has a greater advantage in forward prediction. Additionally, when observing the model performance under different seasonal data, there are large differences in the RMSE and MAE under different seasons, with the worst performance occurring in the winter data and the best performance occurring in the autumn data, which may be related to weather changes in different seasons in the region. Therefore, in many studies, it is necessary to train and test models by month or season.

## Conclusion

To improve the accuracy of PV power prediction and ensure the balance between PV power generation and grid supply and demand, this paper proposes a TCN-GRU neural network model based on the optimization of an efficient channel attention mechanism. This paper uses TCN neural networks to extract spatial features from multiple weather features and photovoltaic power sequences, embeds an ECA attention mechanism after the convolution layer, generates channel weights with the help of one-dimensional convolution to efficiently achieve local cross-channel interaction to capture important information, and then builds time series features through the GRU to output power prediction values. The model was experimentally validated to have the following advantages:

1. TCNs have a larger field of perception through fewer layers than CNNs and can therefore receive longer historical data, making them more suitable than CNNs for multifeature extraction and fusion for PV power forecasting.
2. The ECA module gains the ability to capture important information efficiently by generating channel weights via one-dimensional convolution without dimensionality reduction, which can significantly improve the performance of the TCN.
3. The proposed model generally performs well on the four seasons of data, with a minimum RMSE of 0.0195, a minimum MAE of 0.0128 and a maximum R^2^ of 99.72%, with maximum improvements of 11.32%, 8.57% and 0.38%, respectively, over the suboptimal model.

The model proposed in this paper has promising applications in the field of short-term PV power prediction and can provide highly accurate prediction results.

Although the model proposed in this paper has good performance, several issues remain to be solved: (1) In this study, we consider only single-step prediction and 3-, 6-, and 9-step predictions and predict the power for the next 5 min, 15 min, 30 min and 45 min, respectively. In the future, we can further explore the model's prediction accuracy for the next 1 h, 12 h or even a day to further explore the application of the model in different fields of time series. (2) This study focuses on capturing and processing multivariate features through the characteristics of the temporal convolutional network itself. In the future, we can explore the correlation between the factors affecting power generation in a more in-depth manner to obtain more accurate and stable prediction results. (3) The dataset used to train the models in this study had a 5 min temporal resolution, so future work could attempt to train models with longer-term temporal resolution data to improve model applicability.

## Data Availability

The experimental data used in this paper are from a photovoltaic power dataset (https://dkasolarcentre.com.au/download?location=yulara) from a DKASC photovoltaic power station in Australia.
